# Synonymous Codon Pattern of Cowpea Mild Mottle Virus Sheds Light on Its Host Adaptation and Genome Evolution

**DOI:** 10.3390/pathogens11040419

**Published:** 2022-03-30

**Authors:** Siqi Yang, Ye Liu, Xiaoyun Wu, Xiaofei Cheng, Xiaoxia Wu

**Affiliations:** 1 Key Laboratory of Germplasm Enhancement, Physiology and Ecology of Food Crops in Cold Region of Chinese Education Ministry, College of Agriculture, Northeast Agricultural University, Harbin 150030, China; s190301903@neau.edu.cn (S.Y.); s200301019@neau.edu.cn (Y.L.); xiaoyun.wu@neau.edu.cn (X.W.); 2 Key Laboratory of Soybean Biology of Chinese Education Ministry, College of Agriculture, Northeast Agricultural University, Harbin 150030, China

**Keywords:** cowpea mild mottle virus, codon pattern, dinucleotide bias, codon adaptation, nucleotide composition

## Abstract

*Cowpea mild mottle virus* (CpMMV) is an economically significant virus that causes severe disease on several legume crops. Aside from recombination, other factors driving its rapid evolution are elusive. In this study, the synonymous codon pattern of CpMMV and factors shaping it were analyzed. Phylogeny and nucleotide composition analyses showed that isolates of different geography or hosts had very similar nucleotide compositions. Relative synonymous codon usage (RSCU) and neutrality analyses suggest that CpMMV prefers A/U-ending codons and natural selection is the dominative factor that affects its codon bias. Dinucleotide composition and codon adaptation analyses indicate that the codon pattern of CpMMV is mainly shaped by the requirement of escaping of host dinucleotide-associated antiviral responses and translational efficiency.

## 1. Introduction

*Cowpea mild mottle virus* (CpMMV), a whitefly (*Bemisia tabaci*)-transmitted virus of the genus *Carlavirus* within the family *Betaflexviridae*, has filamentous particles of about 650 nm in length and a diameter of about 13 nm [1]. The genome of CpMMV is composed of a positive-sense, single-stranded RNA (+ssRNA) that is capped at the 5′-end and polyadenylated at the 3′-end. The genome of CpMMV encodes a total number of six open reading frames (ORFs). The largest ORF on the 5′-termini encodes the viral RNA-dependent RNA polymerase (RdRp). ORF2-4 are partially overlapped and are composed of the so-called triple-gene-block (TGB) module, which encodes TGBp1, TGBp2, and TGBp3, respectively. ORF5 encodes the viral coat protein (CP), while ORF6 encodes a small cysteine-rich protein that may function as the pathogenicity factor and RNA silencing suppressor [2,3,4]. ORF1 is translated from genomic RNA, while ORF2-4 and ORF5-6 are translated from two subgenomic RNAs [5].

Although CMMV was thought to be only locally distributed when first found on cowpea (*Vigna unguiculata*) in Ghana [6], it has since been shown to have a worldwide distribution and a wide natural host range, e.g., tomato (*Lycopersicon esculentum*), groundnut (*Arachis hypogaea*), peanut grass (*Arachis repens*), soybean (*Glycine max*), French bean (*Phaseolus vulgars*), cowpea (*Vigna unguiculata*), urdbean (*Vigna mungo* Black gram), mungbean (*Vigna radiata*), Adzuki bean (*Vigna angularis*), Chia (*Salvia hispanica*), and papaya (*Carica papaya*) [7,8,9,10,11,12,13,14,15,16]. Recent outbreaks of CpMMV on soybean in Brazil and China further emphasize its economic importance [17,18]. Studies showed that CpMMV is abundant in genetic diversity, which may be responsible for its wide host range and pathogenicity on soybean [5,14,17]. Moreover, isolates of different origins showed distinct biological properties, suggesting their high genetic flexibility and adaptation [8,9,10]. Indeed, studies showed that recombination plays an important role in the adaptation and symptomology of CpMMV in soybean [18,19]; however, other genetic forces driving the evolution of CpMMV are still elusive.

The genetic information is read by translational machinery in a triple-nucleotide manner from 5′ to 3′, each of which is called a genetic codon that is translated to a special amino acid or as a translation stop codon. Except for three stop codons, there are 61 codons for 20 natural amino acids, resulting in most amino acids being encoded by more than one codon. Codons for the same amino acid are known as synonymous codons. Synonymous codons are not used equally; instead, different species show different codon preferences. Even genes of the same species display differences in codon patterns. Codon usage bias can be affected by many factors, e.g., nucleotide composition, tRNA abundance, mutation bias, natural selection, gene length, and even expression level [20]. Viruses are obligate parasites that rely on host translation machinery for protein translation. As a result, viral codon patterns may be significantly affected by the hosts they are infecting. For instance, rubella virus (RUBV) and citrus tristeza virus (CTV) showed high codon adaptation to their respective hosts [21,22]. The increased nucleotide sequence data of CpMMV from different hosts in recent years allow us to gain detailed insight into its genetic properties, e.g., nucleotide composition, codon pattern, mutation bias, and the influence of host on these genetic properties. Our results showed that the host is an important factor driving the evolution of CpMMV by shaping its dinucleotide composition and codon pattern.

## 2. Materials and Methods

### 2.1. Data Availability

A total number of fifty-five full-length genome or the *Coat protein* (CP) sequences of CpMMV were retrieved from the National Center for Biotechnology Information (NCBI) GenBank nucleotide database. The database also includes the accession number, country of origin, isolate name, and host of each sequence—see Table 1. A total number of 122,218, 41,097, and 25,026 RefSeq mRNA sequences of soybean, cowpea, and papaya were downloaded from the NCBI GenBank nucleotide database.

### 2.2. Phylogenetic Analysis

A phylogenetic tree was constructed using the MEGA 11 software with the neighbor-joining (NJ) algorithm [23]. The nucleotide substitution model, mutation rate, and pattern were determined using the Model Selection function in MEGA 11. The bootstrapped confidence interval was based on 1000 replicates.

### 2.3. Calculation of the Relative Synonymous Codon Usage (RSCU)

RSCU is defined as the ratio of observed to expected codon frequency under equal codon usage without being affected by the amino acid compositions or the CDS sizes of different gene samples [24]. Synonymous codons with RSCU values lower than 1, equal to 1, and higher than 1 represent negative, no, and positive codon usage bias, respectively. Synonymous codons with RSCU values > 1.5 and <0.6 can be recognized as significantly overrepresented and underrepresented codons, respectively [25]. The RSCU of CpMMV was calculated with a homebuilt BioPython script. To calculate the RSCU values of host, open reading frames (ORFs) within mRNA sequences were extracted using a homemade BioPython script, which also removed any incomplete and erroneous sequences. The RSCU values of soybean, cowpea, and papaya were calculated by a BioPython script from a total number of 89,784, 41,014, and 25,430 full-length coding sequences, respectively. RSCU values of soybean leaf-specific and seed-specific genes were derived from [26], which were calculated from 58 and 21 highly expressed leaf-specific and seed-specific genes, respectively. All BioPython scripts are available upon request.

### 2.4. Nucleotide Composition Analysis

The ORFs in each CpMMV genome were retrieved manually and then were aligned by Clustal W 2.0 with default parameters [27]. Overall nucleotide composition (%A, %C, %U, and %G), nucleotide composition at the third codon position (%A3s, %C3s, %U3s, and %G3s), and mean frequencies of G + C nucleotide at the first, second, and third codon position (GC1s, GC2s, and GC3s) were calculated using the CodonW software version 1.4.2 with default parameters, which is available at http://codonw.sourceforge.net (accessed on 1 March 2022).

### 2.5. Analysis of Effective Number of Codons (ENC)

ENC is a simple and efficient method to quantify codon bias independent of gene length and amino acid composition [28]. The value of an ENC analysis ranges from 20 (when only one synonymous codon is used for the corresponding amino acid) to 61 (when all synonymous codons are used equally). The codon usage patterns were investigated by the NEC plot, a plot of NEC vs. GC3s at synonymous sites. In an ENC plot, the observed and expected ENC values are compared to determine the influence of structuring synonymous codon usage bias. An ENC plot is commonly used to determine the effect of G + C compositional constraints on codon usage bias. When the corresponding points fall near the expected curve, the mutation is the main force shaping codon usage. Alternatively, natural selection is the main force shaping codon usage when the corresponding points fall considerably below the expected curve.

### 2.6. Neutrality Plot Analysis

A neutrality plot was used to identify the effects of natural selection and mutation pressure on the codon pattern [29]. In a neutrality plot, the average GC content at GC12s was plotted against GC3s. A regression line was plotted between the GC3s-variable and the GC12s-variable. Unlike GC3s, GC1 and GC2 are subject to functional constraints, because a mutation at these positions usually leads to an amino acid change. As a result, the evolutionary speed of the mutation and natural selection pressure is expressed as the slope of the regression line, while the regression coefficient implies the mutation-selection equilibrium coefficient [30]. Theoretically, the mutation is recognized as the main force shaping the codon pattern when the slope of the regression line is close to 1, while natural selection is accepted as the main force when the slope of the regression line is close to 0.

### 2.7. Dinucleotide Odds Ratio

The dinucleotide odds ratio was calculated as described using a homebuilt BioPython script that is available upon request [31]. Based on the statistical theory, the dinucleotide can be recognized as significantly underrepresented if the ratio is equal or less than 0.78 and significantly overrepresented if the ratio is equal or higher than 1.23 [31].

## 3. Results

### 3.1. Phylogenetic Analysis

**Figure 1:**
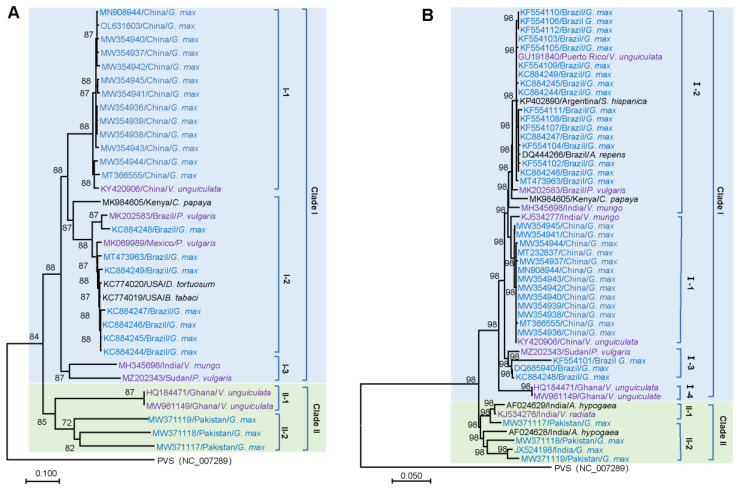
Phylogeny of CpMMV. (**A**) Neighbor-joining phylogenetic tree based on full-genome sequences of CpMMV. (**B**) Neighbor-joining phylogenetic tree of CpMMV based on sequences of *CP* gene. Numbers on branches indicate the percentage support values of 1000 bootstrap replicates. The GenBank accession number, origin, and host of each sequence are indicated. Isolates from soybean, other legumes, and non-legumes are in blue, purple, and black, respectively. Clades I and II are also highlighted in light blue and light green backgrounds, respectively.

To gain insight into the phylogenetic relationship of CpMMV isolates, a neighbor-joining (NJ) phylogenetic tree was constructed using thirty-three full-length genomes of CpMMV (Table 1). Consistent with the previous study [32], the phylogenetic tree was apparently clustered into two major clades, namely Clade I and Clade II (Figure 1A). Clade I could be further separated into three subclades, namely subclades I-1, I-2, and I-3. Subclade I-1 contained thirteen isolates from China; subclade I-2 contained eleven isolates from America and a papaya isolate from Kenya (MK984605); subclade I-3 contained two isolates from India and Sudan, respectively. Clade II could be further divided into two subclades: subclade II-1 contained two isolates from Ghana; subclade II-2 contained three isolates from Pakistan. We further analyzed the phylogenetic relationship of the thirty-three CpMMV isolates based on hosts that were isolated. We found that isolates from soybean and cowpea were located in Clades I and II; isolates from other legumes and non-legume hosts were clustered in Clade I (Figure 1A).

Since recombination is prevalent in CpMMV isolates [17,18,19], which may mask the real phylogenetic relationship of these isolates; therefore, we constructed a phylogenetic tree based on the *CP* gene, which has a low recombination ratio. A total number of 161 sequences of the *CP* gene were retrieved from the GenBank database. For clarity, sequences with higher than 99% nucleotide sequence similarities were removed. A phylogenetic tree was then constructed using the rest 51 sequences of the *CP* gene (Figure 1B). Consistent with the NJ tree based on full-length genomes, the 51 sequences of the *CP* gene were also clustered into two major Clades; however, the two isolates of subclade II-1 in the NJ tree constructed based on the full-length genome was comprised of the fourth subclade in Clade I. Geographic distribution analysis showed that isolates from India display a high degree of diversity since they were present in Clade I and Clade II. In this *CP*-based NJ tree, isolates from soybean were distributed in Clades I and II, isolates from cowpea were located in Clade I, and isolates from other legumes and non-legume hosts were found in Clades I and II (Figure 1B).

### 3.2. Nucleotide Composition Analysis

To gain insight into the genetic forces driving the evolution of CpMMV, we first analyzed the nucleotide composition of the full-length genome of the thirty-three CpMMV isolates (excluding the poly-A tail). Results showed that the overall nucleotide composition of CpMMV was T and U-rich; U and C were the most abundant and infrequent bases, respectively (Table 2). We further calculated the nucleotide composition in the protein-coding regions of the thirty-three CpMMV isolates. The GC1 position had the highest G + C content in the CpMMV coding sequence (0.41), while the GC2 position had the lowest G + C content (0.40). Further analysis of the third wobble codon position revealed a significantly higher abundance of U3 (0.41) and A3 (0.35) than G3 (0.28) and C3 (0.24) (Table 2). These results suggest that CpMMV displays a codon pattern that prefers A/U-ending codons; and, the codon pattern of CpMMV may be affected by compositional constraints. CpMMV had an overall effective number of codons (ENC) value of 52.23, suggesting a moderate but highly conserved codon pattern bias of CpMMV. The variations of nucleotide composition and ENC among isolates of Clade I and Clade II or of hosts were ignorable (Table 2), indicating that different isolates have similar mutation bias and natural selection.

### 3.3. Natural Selection Is the Major Force Influencing the Codon Usage of CpMMV

**Figure 2:**
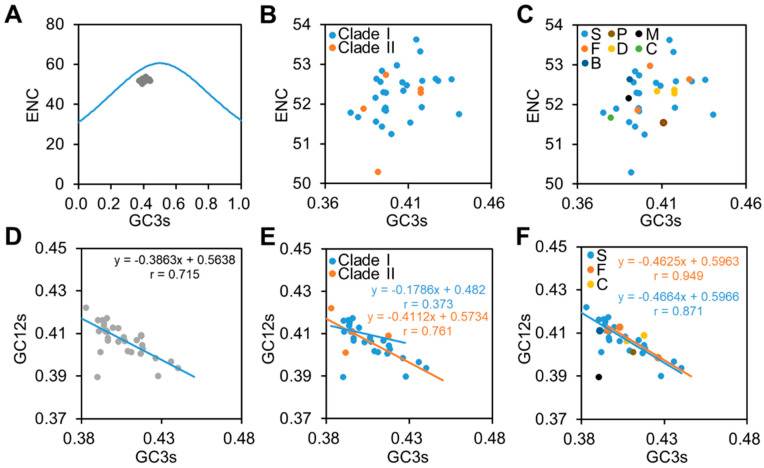
ENC plot and neutrality analysis. (**A**) ENC plot of the thirty-three CpMMV isolates. Blue curve indicates the expected curve when all codons are used randomly (no selection). (**B**,**C**) An enlarged image of the ENC plot to show the distribution of CpMMV isolates according to their phylogenetic relationships (**B**) or hosts (**C**). (**D**–**F**) Correlation between nucleotide composition GC12 and GC3 of all thirty-three isolates (**D**) or according to their phylogenetic relationships (**E**) or hosts (**F**). S, P, M, F, D, C, and B represent soybean, papaya, mungbean, French bean, Florida beggarweed (*D. tortuosum*), cowpea, and whitefly, respectively.

To evaluate the forces shaping the codon pattern patterns of CpMMV, an ENC plot and neutrality analysis were carried out. In the ENC plot, all CpMMV isolates were clustered together and located below the expected curve, suggesting that the codon pattern of CpMMV is subjected to GC compositional constraints (Figure 2A). A detailed survey showed that isolates of Clade I and Clade II of different hosts were distributed in the same area (Figure 2B,C), further confirming that different CpMMV isolates are subjected to similar GC compositional constraints. In the neutrality analysis (Figure 2D), average values of GC3s were negatively correlated with the average values of GC12s (r = 0.715; *p* < 0.01); however, the slope of the regression line was 0.3863, which suggests that mutation pressure accounts for approximately 38.63% of variations and natural selection accounts for 61.37% of variations. Neutrality analysis was also performed according to their phylogenetic relationships and hosts. Results showed that the average values of GC3s were also negatively correlated with the average values of GC12s (Figure 2E,F) and slopes values were closer to 0 than to 1. Thus, natural selection instead of mutation pressure plays a dominant role in shaping the codon pattern of CpMMV.

### 3.4. RSCU Patterns of CpMMV

To gain insight into the codon pattern of CpMMV, we calculated the overall RSCU values of the thirty-three CpMMV isolates (Table 3). Among the 59 synonymous codons, 9 and 12 were identified as significantly preferred codons (RSCU value > 1.5) and unpreferred codons (RSCU value < 0.6), respectively. An analysis of the base composition at the third codon showed that 7 out of 9 significant codons and 24 out of 27 preferred codons (RSCU value > 1.0) were A or U-ending, while 9 out the 12 significantly unpreferred codons were G or C-ending, suggesting that CpMMV prefers A/U-ending codons.

**Figure 3:**
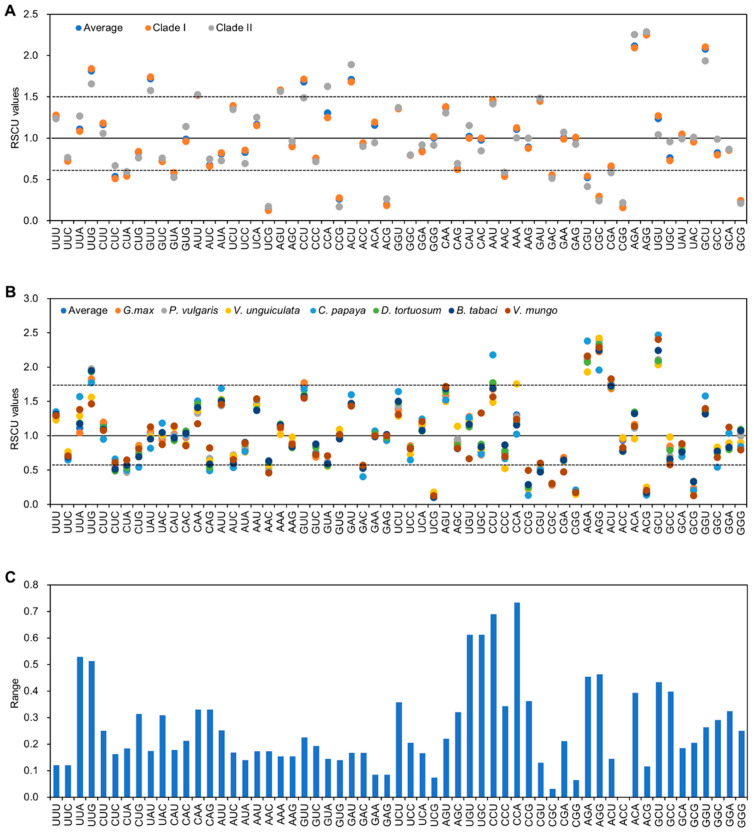
Graphic illustration the variations of CpMMV synonymous codon usage. (**A**) The average RSCU values of CpMMV isolates of Clades I and II. (**B**) The average RSCU values of CpMMV isolates of seven hosts. (**C**) The range of RSCU values of CpMMV isolates of seven hosts.

We further analyzed the RSCU of CpMMV based on their phylogenetic relationship. The average RSCU values of Clades I and II were very close to that of overall RSCU values (Table S1). Especially, the significantly preferred and unpreferred codons of Clades I and II were identical to that of the overall RSCU values (Figure 3A; Table S1). The RSCU values of the thirty-three CpMMV isolates according to their hosts were also calculated (Table S1). Noticeably, RSCU values of several synonymous codons, e.g., CCU, CCU, UGU, UGC, UUA, and UUG, showed high variations (Figure 3B). Specifically, CCA had the highest range (R = 0.734), followed by CCU (R = 0.689), UGU/UGC (R = 0.612), and UUA (R = 0.528) (Figure 3C). The extremum RSCU values were observed between soybean, cowpea, mungbean, and papaya-infecting CpMMV isolates, indicating that the codon pattern of CpMMV may be strongly affected by these hosts.

### 3.5. Dinucleotide Frequency Affects the Codon Pattern of CpMMV

**Figure 4:**
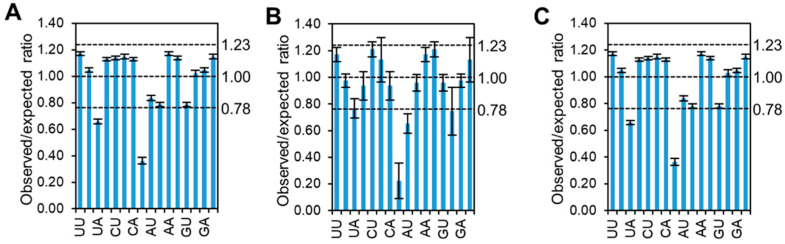
Histogram showing the dinucleotide pattern of CpMMV. (**A**) Overall dinucleotide pattern of CpMMV. (**B**) Dinucleotide pattern of untranslated regions of CpMMV. (**C**) Dinucleotide pattern of coding region of CpMMV.

Since the codon pattern of CpMMV is subject to GC compositional constraint and previous studies showed that several pairs of dinucleotides in RNA viruses were strongly subjected to host selection [31,33], the dinucleotide frequency of CpMMV was analyzed to gain insight into the constraint of dinucleotide bias on the codon pattern. Results showed that CpG and UpA were significantly underrepresented (observed/expected ratio ≤ 0.78) in both the coding region and untranslated regions (UTRs), while no dinucleotide pairs were found to be significantly overrepresented (observed/expected ratio ≥ 1.23) (Figure 4), suggesting that the codon pattern of CpMMV may be affected by CpG and UpA dinucleotide bias. A direct comparison of the RSCU values of codons with an underrepresented dinucleotide showed that the codon pattern is highly affected by the dinucleotide frequency. For instance, four of the six synonymous Arg containing CpG on the first and second codon position (CGN; N indicates A, U, G, or C) were underrepresented (Table 2). Codons containing CpG on the second and third codon position (ACG (coding Thr), UCG (coding Ser), GCG (coding Ala), and CCG (coding Pro)) were also significantly unpreferred (Table 3). Although UpA is contained in two stop codons, we still could find the RSCU values of three out of the four codons containing UpA on the second and third codon position (AUA (coding Ile), GUA (coding Val), and CUA (coding Leu)) were lower than expected (Table 2). 

### 3.6. Codon Adaptation Analysis

**Figure 5:**
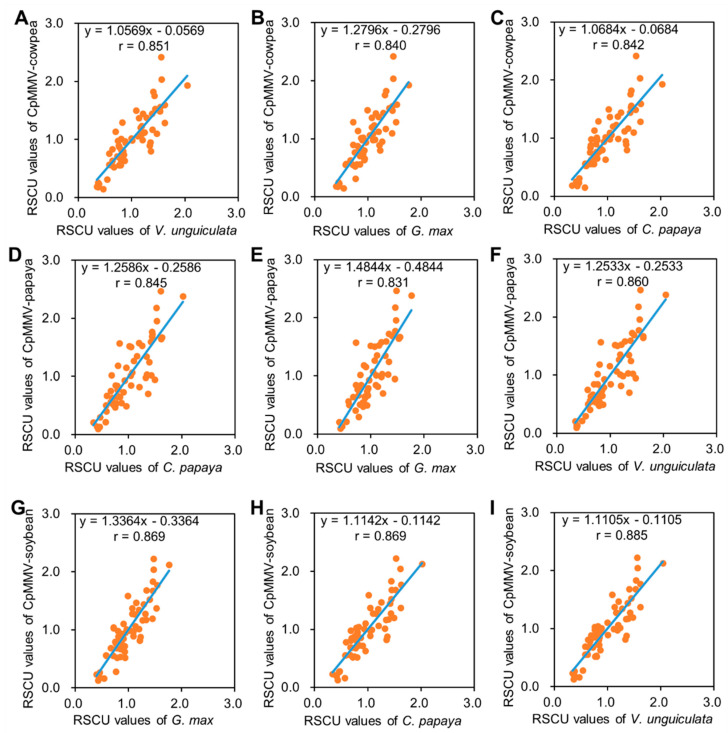
Correlations of the codon abundances of CpMMV and soybean, cowpea, and papaya. (**A**–**C**) RSCU values of cowpea-infecting CpMMV versus overall RSCU values of cowpea (**A**), soybean (**B**), and papaya (**C**). (**D**–**F**) RSCU values of papaya-infecting CpMMV versus RSCU values of papaya (**D**), soybean (**E**), and cowpea (**F**). (**G**–**I**) RSCU values of soybean-infecting CpMMV versus RSCU values of soybean (**G**), papaya (**H**), and cowpea (**I**).

To further gain insight into the impact of the host on CpMMV codon pattern, the overall codon pattern of soybean, cowpea, and papaya were calculated using all available nuclear genes in the GenBank nucleotide database (mungbean was not included due to data availability; Table S2). Corresponding analyses were then performed between RSCU values of CpMMV isolates from soybean (CpMMV-soybean), CpMMV isolates from cowpea (CpMMV-cowpea), or CpMMV isolates from papaya (CpMMV-papaya) and that of soybean, cowpea, and papaya. As expected, the codon pattern of CpMMV-cowpea was most adapted to cowpea according to the correlation coefficient (Figure 5A–C). Interestingly, the codon patterns of CpMMV-papaya and CpMMV-soybean also displayed the highest adaptation to that of cowpea but not papaya or soybean (Figure 5D–I), indicating that cowpea might be the original host of CpMMV. Compared to soybean, the codon pattern of CpMMV-papaya was more similar to that of papaya (Figure 5D,E), suggesting that the infection of papaya causes the readaptation of the codon pattern from cowpea to papaya. The codon pattern of CpMMV-soybean displayed a similar correlation coefficient to the codon pattern of soybean and papaya (Figure 5G–I), implying that papaya and soybean have similar codon usage or CpMMV-soybean possibly originated from papaya.

To further gain insight into the codon adaptation, we compared the codon pattern of CpMMV with that of soybean leaf and seed. It has been reported that the codon usage of genes that were highly expressed in leaf tissues dramatically differed from that of genes expressed in seed [26]; therefore, it is expected that the codon usage of CpMMV is closer to that of leaf-specific genes than seed-specific genes since CpMMV is a phloem-limited virus. As expected, we found that the codon usage of CpMMV is highly correlated to that of leaf-specific genes (r = 0.883; *p* < 0.01), but not seed-specific genes (r = 0.402; *p* > 0.05) (Figure 5); thus, the codon pattern of CpMMV is highly adapted to the host it infects.

## 4. Discussion

In this study, possible forces other than recombination that drive the evolution of CpMMV were analyzed using phylogeny, nucleotide composition, codon pattern, neutrality and ENC plot, dinucleotide bias, and host adaptation. Phylogenetic analyses showed that isolates from Africa and Southwest Asia were present in Clades I and II; isolates from East Asia and America were distributed only in Clade I (Figure 1), suggesting that CpMMV possibly originated from Africa and/or Southwest Asia and was introduced to East Asia and America through the international trade of agricultural products or along with the spread of its vector: silverleaf whitefly (*Bemisia tabaci* MEAM1). The availability of more sequences from other regions of the world could confirm or rule out this hypothesis. Codon adaptation analysis supported the fact that cowpea is the original host of CpMMV, while papaya may be an important intermediate host of CpMMV. Nevertheless, further studies are needed to confirm these possibilities.

Nucleotide composition, codon pattern, and ENC plot neutrality analysis showed that CpMMV had a codon pattern that prefers A/U-ending codons. Neutrality analysis showed that the RSCU of CpMMV is subjected to GC constraint and that natural selection instead of mutation pressure plays a dominant role in shaping the codon pattern of CpMMV. Dinucleotide frequency analysis showed that CpG and UpA were significantly underrepresented in both the untranslational regions and coding regions of CpMMV (Figure 4). Interestingly, codons with underrepresented dinucleotides were always significantly unpreferred (Table 2), suggesting that the RSCU of CpMMV is heavily affected by dinucleotide bias, especially CpG and UpA. The dinucleotide pattern is a stable genomic signature, which is related to genome compartmentalization, gene evolution, and gene expression regulation [34]. Studies showed that UpA is underrepresented in almost all organisms tested, whereas CpG is differentially represented in the genomes of eukaryotic organisms [35]. It was proposed that the TpA depletion may be caused by its presence in two out of three stop codons and in transcriptional regulatory motifs (e.g., the TATA box sequence), while CpG repression has been directly linked to cytosine methylation in DNA [36]. The dinucleotides CpG and UpA are also significantly underrepresented in the genome of retroviruses and riboviruses [31,33,37]. Importantly, increasing or decreasing the CpG and UpA dinucleotide frequencies in the genome could significantly attenuate or increase viral proliferation, respectively [38,39,40]. Recently, it was found that the attenuation of viruses with elevated frequencies of CpG and UpA dinucleotides in animals was associated with the ZAP and OAS3/RNAseL-mediated antiviral pathways, respectively [41,42]. How CpG and UpA affect the codon pattern of plant RNA viruses such as CpMMV is an interesting question for investigation in the future since neither ZAP nor OAS3/RNAseL counterparts have been identified in plants.

**Figure 6:**
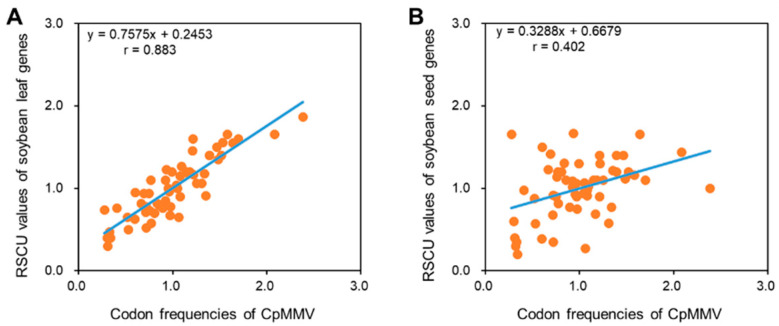
The codon pattern of CpMMV is adapted to leaf-specific genes of soybean. (**A**,**B**) RSCU values of CpMMV versus RSCU values of leaf-specific genes of soybean (**A**) and seed-specific genes of soybean (**B**).

It has been proposed that the translation of mRNA with rare codons is less efficient than those with optimal codons since the latter have more cognate tRNA species [43]. As a result, a higher translation efficiency certainly will benefit the proliferation of viruses. Indeed, studies showed that the codon pattern of many viruses is highly adapted to their hosts [21,44,45]. Our results also showed that the codon pattern of CpMMV was overall highly adapted to its hosts, e.g., cowpea, papaya, and soybean. Moreover, our results showed that the codon pattern of soybean-isolated CpMMV and papaya-isolated CpMMV was closer to that of soybean and papaya, respectively (Figure 5), which further confirmed the impact of the host on the codon pattern of CpMMV. Moreover, the codon usage of CpMMV resembles the codon usage of leaf-specific genes but not that of seed-specific genes (Figure 6). Thus, mimicking the host codon pattern for maximum translation efficiency is also an important force shaping the codon pattern of CpMMV.

## 5. Conclusions

The synonymous codon pattern of CpMMV is shaped by the host it infects, possibly through pressures from dinucleotide bias and translational machinery.

## Figures and Tables

**Table 1:** The information of CpMMV isolates used in this study.

No.	GenBank Accession No	Country	Isolate Name	Host
1	AF024628	India	CPMMV-H	*Arachis hypogaea*
2	AF024629	India	CPMMV-M	*A. hypogaea*
3	DQ444266	Brazil	Arachis	*A. repens*
4	DQ885940	Brazil	Barreiras	*Glycine max*
5	GU191840	Puerto Rico	CpMMVPR	*Vigna unguiculata*
6	HQ184471	Ghana	Ghana	*V. unguiculata*
7	JX524198	India	D1	*G. max*
8	KC774019	USA	Whiteflies_2007	*Bemisia tabaci*
9	KC774020	USA	Florida_Bean_2011	*Desmodium tortuosum*
10	KC884244	Brazil	CPMMV:BR:MG:09:2	*G. max*
11	KC884245	Brazil	CPMMV:BR:MG:09:3	*G. max*
12	KC884246	Brazil	CPMMV:BR:MT:02:1	*G. max*
13	KC884247	Brazil	CPMMV:BR:BA:02	*G. max*
14	KC884248	Brazil	CPMMV:BR:GO:01:1	*G. max*
15	KC884249	Brazil	CPMMV:BR:GO:10:5	*G. max*
16	KF554101	Brazil	BR:GO:10:4	*G. max*
17	KF554102	Brazil	BR:MA:02	*G. max*
18	KF554103	Brazil	CPMMV:BR:MG:09:1	*G. max*
19	KF554104	Brazil	CPMMV:BR:MG:09:4	*G. max*
20	KF554105	Brazil	CPMMV:BR:MG:09:5	*G. max*
21	KF554106	Brazil	CPMMV:BR:MG:09:6	*G. max*
22	KF554107	Brazil	BR:MG:09:7	*G. max*
23	KF554108	Brazil	BR:MG:09:11	*G. max*
24	KF554109	Brazil	BR:MG:09:12	*G. max*
25	KF554110	Brazil	BR:MG:09:15	*G. max*
26	KF554111	Brazil	BR:MG:09:16	*G. max*
27	KF554112	Brazil	BR:PA:02	*G. max*
28	KJ534276	India	MUNGBEAN1	*V. radiata*
29	KJ534277	India	URDBEAN1	*V. mungo*
30	KP402890	Argentina	MA01	*Salvia hispanica*
31	KY420906	China	Hainan1	*V. unguiculata*
32	MH345698	India	CpMMV-Urd-Kanpur	*V. mungo*
33	MK069989	Mexico	CN2	*Phaseolus vulgaris*
34	MK202583	Brazil	CPMMV:BR:GO:14	*P. vulgaris*
35	MK984605	Kenya	KE-Kit_01	*Carica papaya*
36	MN908944	China	Anhui_SZ_DN1383	*G. max*
37	MT232837	China	cpmmv-anhui-sz	*G. max*
38	MT366555	China	CPMMV-JS	*G. max*
39	MT473963	Brazil	Casa Branca_BR	*G. max*
40	MW354936	China	CPMMV_HN_LH	*G. max*
41	MW354937	China	CPMMV_SD_JX	*G. max*
42	MW354938	China	CPMMV_HN_XC	*G. max*
43	MW354939	China	CPMMV_HB_JZ	*G. max*
44	MW354940	China	CPMMV_HN_SQ	*G. max*
45	MW354941	China	CPMMV_SD_JN	*G. max*
46	MW354942	China	CPMMV_JL_GZL	*G. max*
47	MW354943	China	CPMMV_JL_CC	*G. max*
48	MW354944	China	CPMMV_JS_NJ	*G. max*
49	MW354945	China	CPMMV_AH_FY	*G. max*
50	MW371117	Pakistan	PK1	*G. max*
51	MW371118	Pakistan	PK2	*G. max*
52	MW371119	Pakistan	PK3	*G. max*
53	MW961149	Ghana	DSMZ PV-0090	*V. unguiculate*
54	MZ202343	Sudan	DSMZ PV-0907	*P. vulgaris*
55	OL631603	China	AH-FY	*G. max*

**Table 2:** The nucleotide composition and properties of CDS of the CpMMV genomes.

Catalogs	Overall	Clade-I	Clade-II	Soybean	Cowpea	French Bean	Mung Bean	Papaya
A	0.29	0.29	0.30	0.29	0.30	0.29	0.30	0.30
U	0.30	0.30	0.29	0.30	0.29	0.30	0.30	0.31
C	0.18	0.18	0.18	0.18	0.18	0.18	0.18	0.17
G	0.23	0.23	0.23	0.23	0.23	0.23	0.23	0.22
GC	0.41	0.41	0.41	0.41	0.41	0.41	0.40	0.39
GC1	0.41	0.41	0.42	0.42	0.41	0.42	0.39	0.39
GC2	0.40	0.40	0.40	0.40	0.41	0.39	0.41	0.39
GC12	0.41	0.41	0.41	0.41	0.41	0.41	0.40	0.39
GC3	0.40	0.40	0.40	0.40	0.41	0.41	0.41	0.39
A3	0.35	0.34	0.36	0.35	0.35	0.34	0.34	0.36
U3	0.41	0.41	0.40	0.41	0.39	0.41	0.41	0.41
C3	0.24	0.24	0.24	0.24	0.24	0.25	0.24	0.22
G3	0.28	0.28	0.27	0.28	0.28	0.28	0.29	0.28
ENC	52.23	52.28	51.91	52.22	52.33	52.49	51.54	52.16

**Table 3:** Relative synonymous codon usage (RSCU) values of CpMMV.

AA	Codon	Overall	AA	Codon	Overall	AA	Codon	Overall
Phe	UUU	1.27	Pro	**CCU**	**1.68**	Glu	GAA	1.00
	UUC	0.73		CCC	0.75		GAG	1.00
Leu	UUA	1.11		CCA	1.31	Arg	CGU	0.52
	**UUG**	**1.81**		CCG	0.26		CGC	0.29
	CUU	1.16	Thr	**ACU**	**1.71**		CGA	0.65
	CUC	0.53		ACC	0.94		CGG	0.17
	CUA	0.55		ACA	1.16		**AGA**	**2.12**
	CUG	0.83		ACG	0.20		**AGG**	**2.25**
Val	**GUU**	**1.72**	Gly	GGU	1.36	Cys	UGU	1.24
	GUC	0.72		GGC	0.79		UGC	0.76
	GUA	0.57		GGA	0.85	Tyr	UAU	1.04
	GUG	0.99		GGG	1.00		UAC	0.96
Ser	UCU	1.39	Gln	CAA	1.37	Ala	**GCU**	**2.08**
	UCC	0.83		CAG	0.63		GCC	0.83
	UCA	1.17	His	CAU	1.02		GCA	0.86
	UCG	0.13		CAC	0.98		GCG	0.24
	**AGU**	**1.58**	Asn	AAU	1.46	Ile	**AUU**	**1.52**
	AGC	0.91		AAC	0.54		AUC	0.67
Lys	AAA	1.11	Asp	GAU	1.45		AUA	0.81
	AAG	0.89		GAC	0.55			

Note: Significantly preferred codons (RSCU value > 1.5) and unpreferred codons (RSCU value < 0.6) are in bold and underlined, respectively.

## Data Availability

Data are contained within the article or Supplementary Material.

## Supplementary Materials

The following supporting information can be downloaded at: https://www.mdpi.com/article/10.3390/pathogens11040419/s1, Table S1: RSCU values of CpMMV; Table S2: RSCU values of hosts.
